# 

**Published:** 1811-04

**Authors:** 


### MONTHLY CATALOGUE OF MEDICAL BOOKS.

An Essay on some of the Stages of the Operation of Cutting for the Stone; illustrated with an Engraving. By C. B. Trye, F.R.S. Callow.

The Anatomy of the Human Body, containing the Anatomy of the Bones, Muscles, Joints, Heart, and Arteries. By John Bell; and that of the Brain and Nerves, the Organs of the Senses, and Viscera; by Charles Bell. Third Edition, in 3 vols. 8vo. Longman and Co.

Outlines of a Course of Lectures on Chemistry, applied to the Arts and Manufactures; delivered in the Theatre of the Surry Institution. By F. Accum. 12mo. Kearsley.

### NOTICES TO CORRESPONDENTS.

A mistake respecting the insertion of the Engravings, which occurred in the preceding month, our Readers will find corrected in this Journal. The Engraving illustrative of the "Case of morbid Enlargement of the Clitoris," which should have accompanied that Case, was overlooked, and that of Eagland's Truss inserted in its place.

We acknowledge Communications from Dr. Clough, Professor of Midwifery; Apothecarius; Mr. Andrews, of Madeira, by Dr. Adams, with Drawings; Mr. Barlow, of Blackburne, &c. &c.

The heavy expence of postage compels us, though unwillingly, to request our Correspondents will frank their letters.

*Printed by E. Hemsted, Great New Street, Fetter Lane.*

